# PARTNERING IN GERIATRICS WORKFORCE ENHANCEMENT PROGRAMS: MODELS TO ENHANCE COLLABORATION AND ENGAGEMENT

**DOI:** 10.1093/geroni/igz038.2972

**Published:** 2019-11-08

**Authors:** Leland Waters, Nina Tumosa

**Affiliations:** 1 Virginia Commonwealth University, Richmond, Virginia, United States; 2 Health Resources and Services Administration, Rockville, Maryland, United States

## Abstract

To achieve their healthcare system transformational goals to improve care for older adults, Geriatrics Workforce Enhancement Programs (GWEPs) facilitate the building of strong relationships among academia, community-based organizations, and primary care networks. Each GWEP develops strategies to formalize collaborations and build sustainable networks to meet program goals while addressing partner needs. Unique models from four GWEPs addressing stakeholder engagement are described, and factors facilitating collaboration are explored. One GWEP achieves mutual goals by collaborating with statewide coalitions that have a history of successful partnerships. Another GWEP achieves programmatic goals through an “all-in” interprofessional model called the Plenary. A third GWEP has capitalized on a shared complex outcome that requires multi-level stakeholder engagement to support aging in place. The final GWEP has coopted the resource exchange model as a conceptual foundation in order to enhance collaboration. Themes emerging from these four models include: (1) the enhancement of interpersonal relationships through communication, trust, and engagement; (2) the importance of commitment to the overall partnership itself; (3) the critical component of resource sharing and synergy across projects; and (4) strategies for sustainability in the face of changes and challenges across healthcare systems. Given the complex nature of person-centered interventions in geriatrics, it truly takes a village to develop and provide services for a heterogeneous, targeted population. This symposium emphasizes key elements of the structures and processes of these transformational GWEP villages.

